# Supplementary material for: Anatomical and functional organization of the human substantia nigra and its connections
Source: eLife. 2017 Aug 21;6:e26653. doi: 10.7554/eLife.26653 (PMC5606848; doi:10.7554/eLife.26653)
Supplement: Transparent reporting form [file elife-26653-transrepform.docx]

***eLife’s* transparent reporting form**

We encourage authors to provide detailed information *within their submission* to facilitate the interpretation and replication of experiments. If you have any questions, please contact us: [editorial@elifesciences.org](mailto:editorial@elifesciences.org).

**Sample-size estimation**

- You should state whether an appropriate sample size was computed when the study was being designed
- You should state the statistical method of sample size computation and any required assumptions
- If no explicit power analysis was used, you should describe how you decided what sample (replicate) size (number) to use

Please outline where this information can be found within the submission (e.g., page numbers or figure legends), or explain why this information doesn’t apply to your submission:

120 subjects for connectivity-based parcellation of SN (Page 6)

430 subjects for correlating anatomical connectivity with impulsivity measures (Page 6)

485 subjects for correlating brain activation with impulsivity measures (Page 5)

We did not design the Human Connectome Project and therefore did not perform pre-study power calculations, however we used all the data available at the time of study design.

The 120 subjects used for connectivity-based parcellation of one brain area is to our knowledge the largest ever used. We used a split half procedure to confirm our findings.

**Replicates**

- You should report how often each experiment was performed
- You should include a definition of biological versus technical replication
- The data obtained should be provided and sufficient information should be provided to indicate the number of independent biological and/or technical replicates
- If you encountered any outliers, you should describe how these were handled
- Criteria for exclusion/inclusion of data should be clearly stated
- High-throughput sequence data should be uploaded before submission, with a private link for reviewers provided (these are available from both GEO and ArrayExpress)

Please outline where this information can be found within the submission (e.g., page numbers or figure legends), or explain why this information doesn’t apply to your submission:

Parcellation of SN was replicated in two independent groups of 60 subjects (Page 6)

A split-half procedure was also used within each group to calculate the replicability of parcellation (Page 9)

**Statistical reporting**

- Statistical analysis methods should be described and justified
- Raw data should be presented in figures whenever informative to do so (typically when N per group is less than 10)
- For each experiment, you should identify the statistical tests used, exact values of N, definitions of center, methods of multiple test correction, and dispersion and precision measures (e.g., mean, median, SD, SEM, confidence intervals; and, for the major substantive results, a measure of effect size (e.g., Pearson's r, Cohen's d)
- Report exact p-values wherever possible alongside the summary statistics and 95% confidence intervals. These should be reported for all key questions and not only when the p-value is less than 0.05.

BOLD response to value and salience was estimated by one-sample T-test and corrected for multiple comparisons using Threshold-Free Cluster Enhancement (TFCE) method (Figure 6-Figure Supplement 1 and 2)

Partial Least Squares with bootstrap resampling was used to generate Figure 7.

Pearson correlation between brain activity and impulsivity measures was reported along with the p-value (Figure 7 Supplement 1).

Please outline where this information can be found within the submission (e.g., page numbers or figure legends), or explain why this information doesn’t apply to your submission:

(For large datasets, or papers with a very large number of statistical tests, you may upload a single table file with tests, Ns, etc., with reference to page numbers in the manuscript.)

**Additional data files (“source data”)**

- We encourage you to upload relevant additional data files, such as numerical data that are represented as a graph in a figure, or as a summary table
- Where provided, these should be in the most useful format, and they can be uploaded as “Source data” files linked to a main figure or table
- Include model definition files including the full list of parameters used
- Include code used for data analysis (e.g., R, MatLab)
- Avoid stating that data files are “available upon request”

Please indicate the figures or tables for which source data files have been provided:

The data used in this study is obtained from a public resource (Human Connectome Project, <http://www.humanconnectome.org/data/> ).

The additional results including parcellation map of SN, whole-brain tractography maps and brain activation maps will be shared online on github or NeuroVault once this paper is accepted. The code for data analysis is not ours but almost all of it is freely available and cited in the paper.
